# Population Pharmacokinetics of Palbociclib in a Real-World Situation

**DOI:** 10.3390/ph14030181

**Published:** 2021-02-24

**Authors:** Bernard Royer, Courèche Kaderbhaï, Jean-David Fumet, Audrey Hennequin, Isabelle Desmoulins, Sylvain Ladoire, Siavoshe Ayati, Didier Mayeur, Sivia Ilie, Antonin Schmitt

**Affiliations:** 1 Laboratoire de Pharmacologie Clinique et Toxicologie, CHU Besançon, 25000 Besançon, France; 2 INSERM, EFS BFC, UMR1098, RIGHT, Interactions Greffon-Hôte-Tumeur/Ingénierie Cellulaire et Génique, University of Bourgogne Franche-Comté, 25000 Besançon, France; 3 Oncology Department, Centre Georges-François Leclerc, 21000 Dijon, France; cgkaderbhai@cgfl.fr (C.K.); jdfumet@cgfl.fr (J.-D.F.); ahennequin@cgfl.fr (A.H.); IDesmoulins@cgfl.fr (I.D.); sladoire@cgfl.fr (S.L.); sayati@cgfl.fr (S.A.); dmayeur@cgfl.fr (D.M.); silie@cgfl.fr (S.I.); 4 INSERM U1231, University of Burgundy Franche-Comté, 21000 Dijon, France; aschmitt@cgfl.fr; 5 Pharmacy Department, Centre Georges-François Leclerc, 21000 Dijon, France

**Keywords:** palbociclib, population pharmacokinetics, real-world situation

## Abstract

Palbociclib is an oral cyclin-dependent kinase inhibitor that is used in combination with aromatase inhibitors in the treatment of postmenopausal women with metastatic breast cancer. Its metabolism profile is associated with an important interpatient variability. We performed a population pharmacokinetics study of palbociclib in women routinely followed in a cancer center. One hundred and fifty-one samples were analyzed. The sampling times after administration ranged from 0.9 to 75 h and the samples were taken between 1 and 21 days after the beginning of the palbociclib cycle. Palbociclib was determined using a validated mass spectrometry method. The best model that described the concentrations was a one-compartment model with first-order absorption and an absorption lag time. Interindividual variability could only be estimated on the clearance and the first-order absorption. Creatinine clearance was found to be a significant covariate for the apparent clearance. No significant covariates could be observed with the first-order absorption. First-order absorption and absorption lag times were difficult to assess because of the constraints linked to the real-world setting due to the small number of samples used during the absorption process. However, palbociclib apparent clearance was satisfactorily estimated. Population pharmacokinetics (POP PK) with palbociclib could help to optimize dosing.

## 1. Introduction

Palbociclib is an oral inhibitor of cyclin-dependent kinase 4 and 6 (CDK4/6). It was first approved in 2015 by the Food and Drug Administration and the European Medicines Agency after a pivotal trial in postmenopausal women with estrogen-positive, human epidermal growth factor receptor 2-negative (HER2-) advanced breast cancer [1]. In this study, the palbociclib plus letrozole displayed a longer median PFS (24.8 months) than the placebo plus letrozole (14.5 months). Further expansions of approval were granted for the treatment of hormone-positive (HR+), HER2-metastatic breast cancer (2016); HR+, HER2-metastatic breast cancer in first-line (2017); and men with HR+, HER2-metastatic breast cancer (2019). The recommended dose of palbociclib is 125 mg once daily per os for 21 consecutive days followed by 7 days off for a complete cycle of 28 days.

After administration, the maximum concentration is generally observed between 6 and 12 h with a bioavailability of palbociclib of 46% [2]. Its binding to human plasma protein is approximately 85% and its mean apparent volume of distribution is 2583 L [2]. The mean (± standard deviation) plasma elimination half-life is 29 (±5) hours in patients with advanced breast cancer, and the steady state is achieved within 8 days following repeated once-daily dosing [2]. Palbociclib is extensively metabolized, mainly via cytochrome P450 (CYP) 3A4 and sulfotransferase 2A1 [2]. Fasted conditions greatly decrease the absorption and exposure of palbociclib. Taken together, these characteristics are in favor of a great variability of its pharmacokinetics (PK), as shown by the 41% to 59% variability for trough concentrations or the 29% to 42% variability for the area under the curve [3].

Paradoxically, while several articles have been published displaying mixed pharmacokinetic–pharmacodynamic models [4,5,6], the population PK (POP PK) model of palbociclib was only published as a poster [7]. The model built by the authors was a two-compartment model with a first-order rate of absorption (K_a_) and a lag time for absorption (T_lag_). To our knowledge, only the latter describes the covariates that partly explain the palbociclib PK parameters’ variability: the food on lag time and bioavailability, the age and body weight on apparent clearance (CL/F) and the body weight on the peripheral volume of distribution. These studies were performed with samples from either drug registration studies or studies intended for a special purpose (renal impairment, for example). In these studies, many samples were taken from each patient and the collection of clinical data was intensive for the assessment of covariates. Based on a limited number of samples per patient and a less exhaustive number of covariates in the real-life settings of routine patient follow-up, we wanted to confirm the impact of the different covariates in such a situation.

## 2. Results

**Figure 1:**
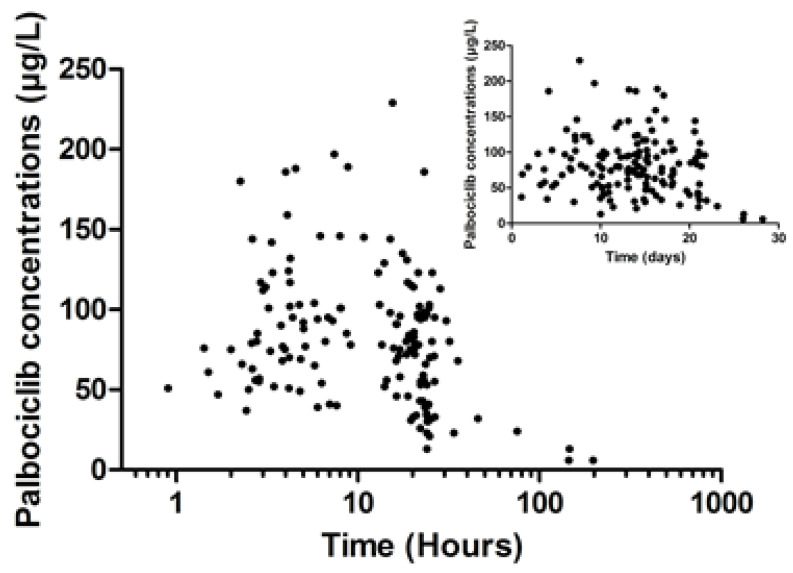
Distribution of palbociclib concentrations (n = 151) over time after administration. With the exception of two patients with two samples, only one sample was drawn per patient. The distribution of the palbociclib concentrations after the beginning of the cycle is displayed as a thumbnail on the graph.

One hundred and fifty-one samples from 124 patients were available for model building. Two samples were taken for 27 patients, but these samples were taken during the same cycle for only two patients. For the other patients, the two samples were taken during different cycles. Thus, no variability between occasions was calculated, and the patients for whom the samples were taken at different cycles were considered as independent. The samples were taken between 1 and 21 days after the beginning of the cycle. Among those, 30 samples (19.9%) were taken during the first 8 days. The samples were taken between 0.9 and 197.25 h after the previous palbociclib administration (Figure 1). The mean concentration was of 81.8 µg/L, while the concentrations ranged from 6 (the LLOQ—lower limit of quantification) to 226 µg/L. No concentrations were below the LLOQ. The administered doses were 75 mg (n = 15), 100 mg (n = 33) and 125 mg (n = 101) per day.

All the patients were women. The patients’ characteristics are displayed in Table 1.

A one-compartment model with a first-order absorption (K_a_), an absorption lag time (T_lag_) and a combined error model was the best model to describe the data. During the model development, no interindividual variability (IIV) could be determined for the apparent central volume of distribution (V/F) or the T_lag_ because it was too small in both cases. Moreover, the precision of the absorption parameters (K_a_ and T_lag_) was not very satisfactory. We then tried to fix one of these parameters to the value that was previously described in the literature [7]. Fixing the K_a_ increased both the Bayesian information criterion (BIC) and the objective function value (OFV), while fixing the T_lag_ led to a decrease of the BIC of 5.0 points together with an improvement of the model in terms of the precision of the parameters, while the OFV was constant. We then decided to fix the T_lag_ to the already published value [7].

**Figure 2:**
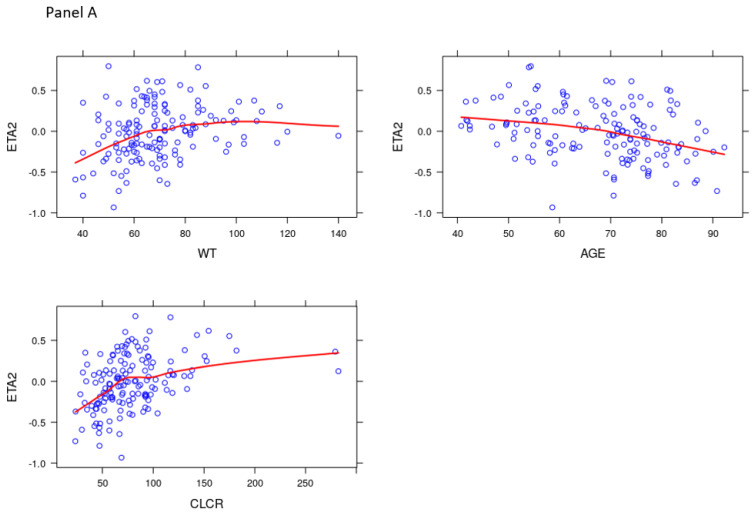

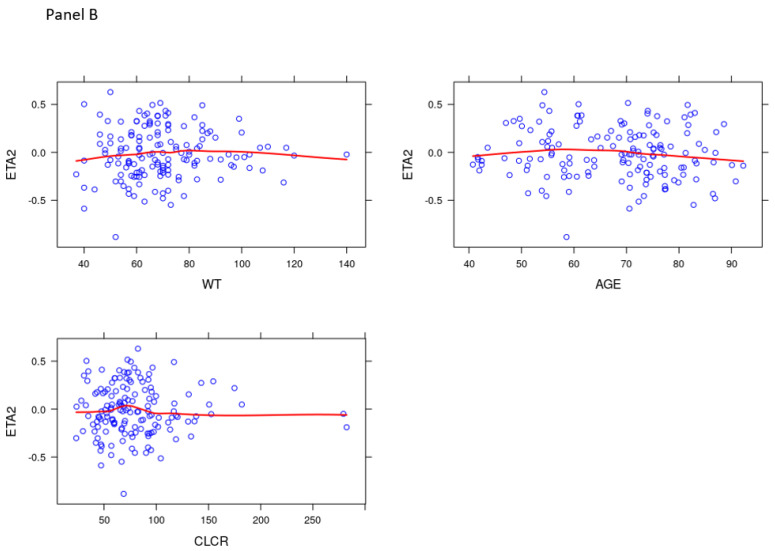
Evolution of the random effect (ETA) associated with the apparent clearance (CL/F) of palbociclib vs. the studied covariates: total body weight (WT), age and creatinine clearance (CLCR). Panel (**A**) was obtained before the addition of CLCR as the covariate to CL/F and panel (**B**) represents the same graphs after the addition of only CLCR as the covariate to CL/F.

**Figure 3:**
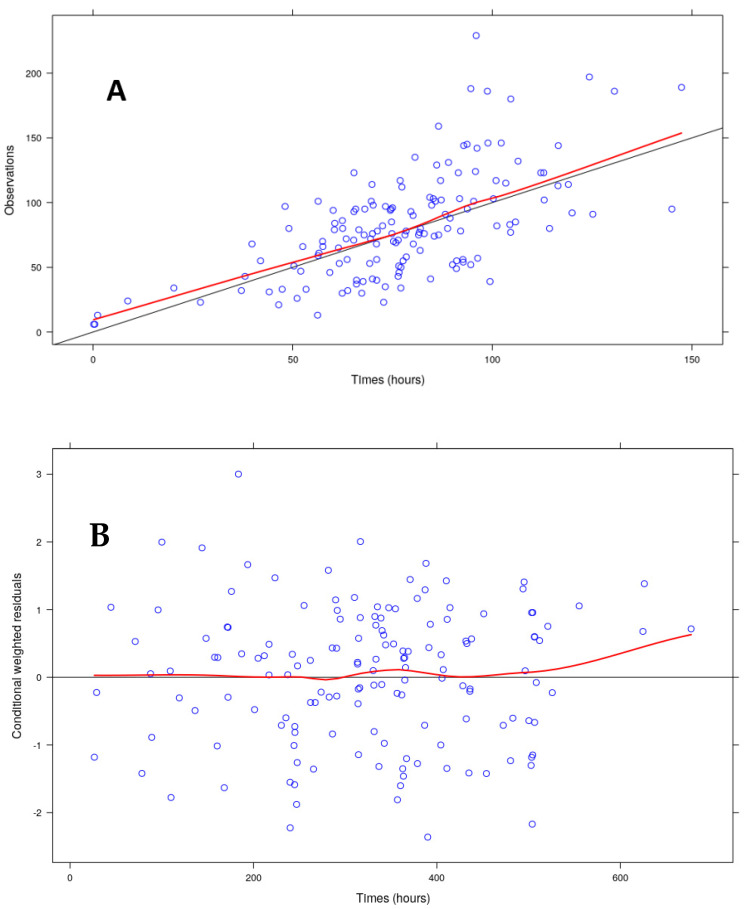

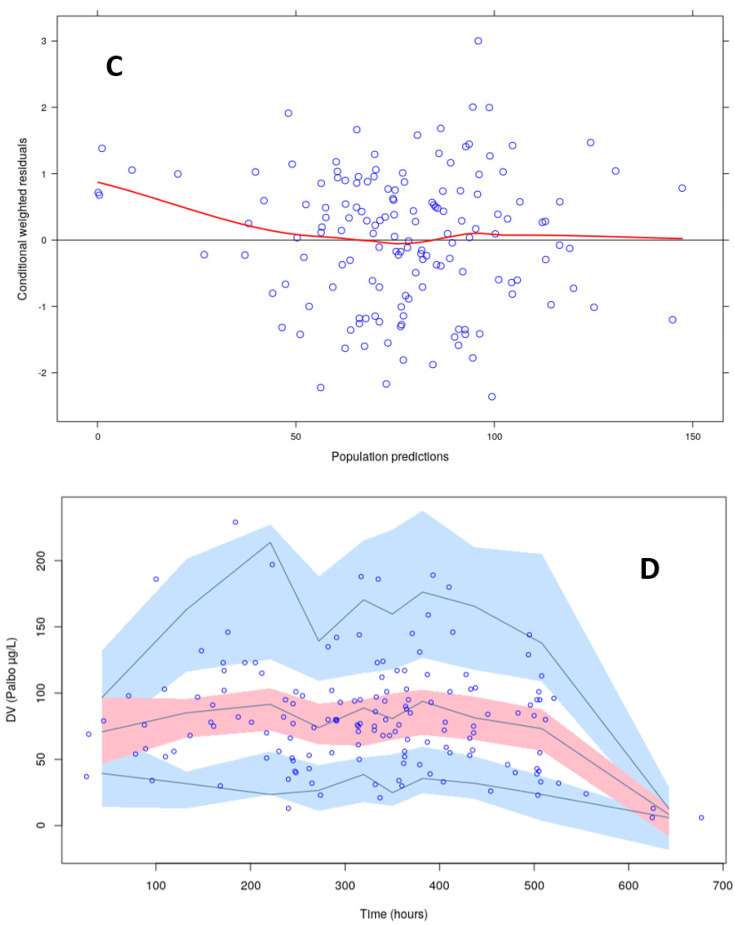

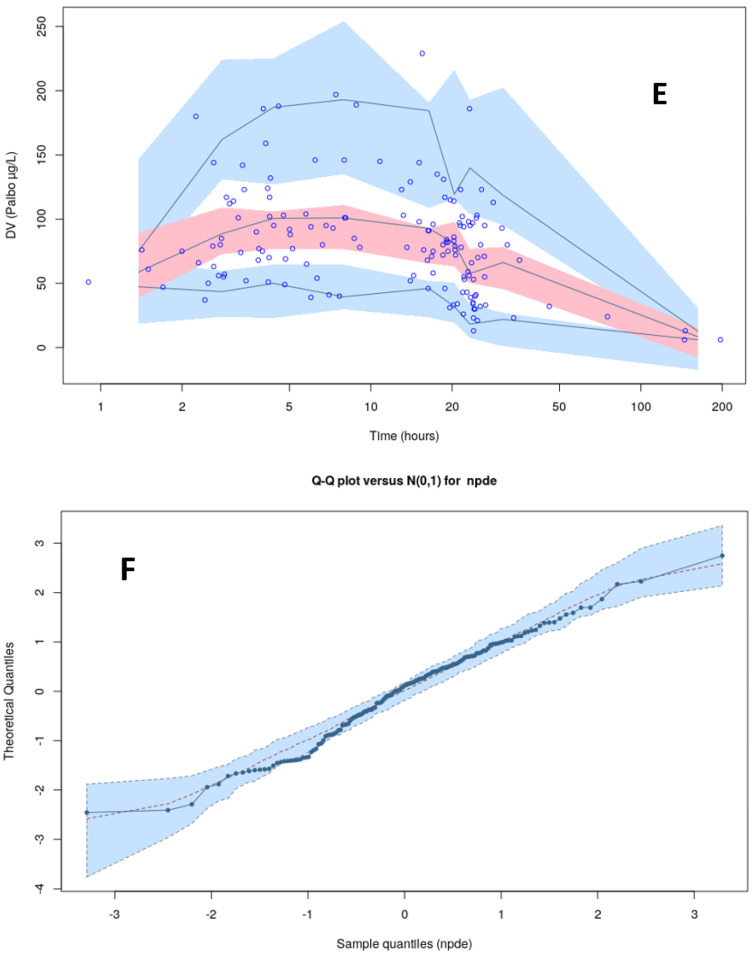

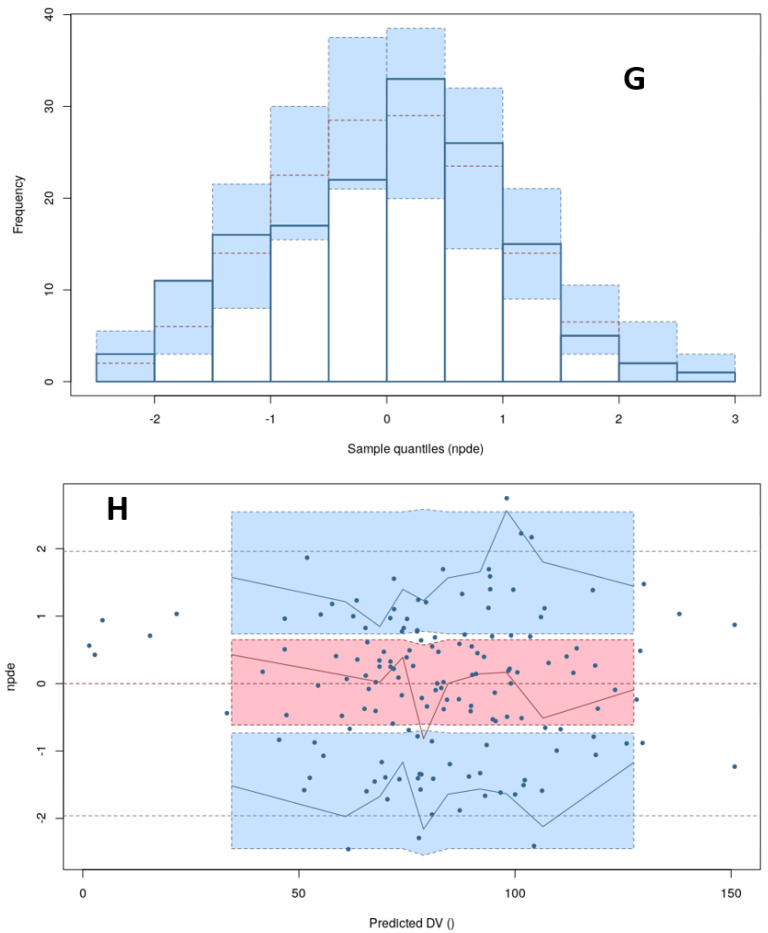

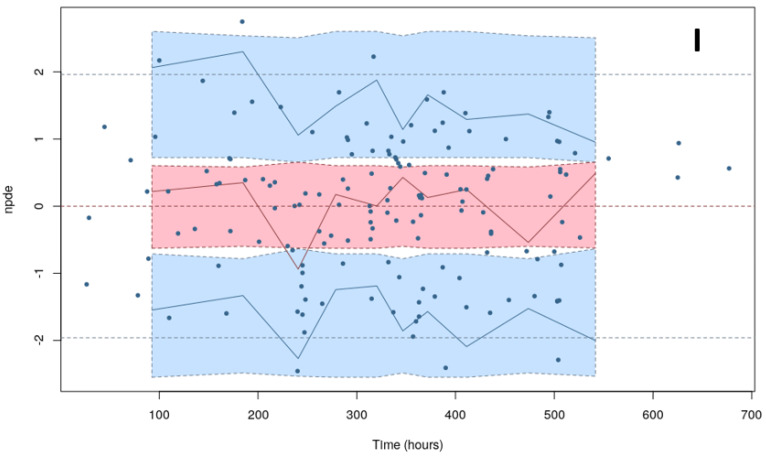
Goodness of fit of the model: (**A**) the observed concentrations (OBS) are plotted vs. the population-predicted concentrations (PRED). (**B**) The conditioning weighted residuals (CWRES) are plotted vs. time, or (**C**) vs. the population-predicted concentrations (PRED). The red line corresponds to the running median of the data and the black line to the identity line (in graphs A, B and C). (**D**) The visual predictive check obtained after 500 simulations with the final model and (**E**) with the data plotted with the time after the dose. In panels D and E: The solid lines represent the running median of the observations. Each line is represented with its CI95 (blue boxes for 2.5th and 97.5th percentiles and pink for the simulated median). The actual concentrations are represented as blue circles. Normalized prediction distribution errors of the final model obtained after 500 simulations. The graphs respectively represent: panel (**F**)—qqplot (plot of the normalized prediction distribution errors (NPDE) vs. the corresponding quantiles of a normal distribution), panel (**G**)—histogram of NPDE together with the normal N(0, 1) distribution, panel (**H**)—NPDE vs. PRED and panel (**I**)—NPDE vs. time.

None of the studied covariates affected the variability of K_a_. However, the addition of serum creatinine, creatinine clearance (CRCL), total body weight (WT) and age led to a decrease of the OFV of 7.74, 32.8, 13.5 and 14.6 points, respectively. These decreases were associated with a decrease of the palbociclib IIV CL/F of 2.2%, 22.7%, 18.9% and 8.3%, respectively. Taking into account the low improvement of the palbociclib IIV CL/F due to the serum creatinine, only the CRCL, WT and age were kept for the backward deletion step. After the application of the backward deletion process, only the CRCL was kept. Neither the associated fulvestrant nor aromatase inhibitors affected the palbociclib clearance. With this model, a correlation was observed between the K_a_ and CL/F. These data were confirmed by an improvement of the random effect (ETA) vs. covariate graphs (Figure 2). Of note, a decrease in the trend observed between this ETA and the WT or age was observed even if CRCL was the only covariate used. The values of the final parameters and results of the bootstrap assessment are displayed in Table 2. The goodness of fit plots of the final model, the visual predictive check (VPC) simulation and the graphs of the normalized prediction distribution errors (NPDE) evaluation are displayed in Figure 3, respectively. Notably, an important shrinkage was observed, especially on K_a_ IIV.

## 3. Discussion

In this study, we developed a POP PK model of palbociclib with only one sample per patient, obtained in a routine setting. In such a situation, we observed difficulties to easily model the absorption. Regarding the absorption, because the maximal concentration of palbociclib extends over 6 h, between 6 h and 12 h after its administration [2], the estimation of both the K_a_ and T_lag_ with only one sample per patient was rendered difficult. Thus, we had to fix the T_lag_ to a value obtained with richer data. Despite this, an important shrinkage was associated with the K_a_ estimation, and its IIV was very important in our hands. However, this was not surprising because even with a very large number of samples, including samples scheduled to assess palbociclib absorption [7], the IIV was previously estimated to be 83.6%.

In the latter study [7], data were obtained from drug registration studies, including a total of 1933 PK observations from 183 patients. Several samples (including full PK sampling schedules) were taken for almost all the patients (251 over 268) during the same or different cycles. With such a large number of samples, the model developed by the authors was a two-compartment model with a first-order rate of absorption K_a_, and a T_lag_. We were only able to describe a one-compartment model. This difference may be explained by the fact that, with patients in routine setting, only one sample was obtained for each patient during a single cycle (except for two patients). Moreover, the great majority of the samples were taken the same day or the day after the administration of palbociclib (i.e., within 36 h after the palbociclib administration), depending on the medical visit schedule. Only four samples were obtained beyond the day after the administration of palbociclib. In such a context, we did not have enough information to develop a two-compartment model as previously described [4,7]. Despite this difference, the value of CL/F we obtained was close to that already published (i.e., CL/F = 58.3 L/h in our study vs. 60.2 L/h).

In the previous mentioned POP PK study [7], both age and body weight were covariates for the CL/F. Even if the baseline values for creatinine clearance and serum creatinine were also assessed as covariates by these authors, they were not retained. In our hands, the body weight, age and creatinine clearance (CRCL) calculated following the Cockroft–Gault formula were selected as covariates for the CL/F. The serum creatinine selection led to a significant decrease of the OFV, but to a very low decrease of the IIV of CL/F, leading to its abandon as a potential covariate. As the CRCL obtained with the Cockroft–Gault formula includes body weight, age and serum creatinine, this was the first covariate we kept during the backward elimination process. Both age and body weight were then eliminated. It was possible to proceed differently: both these covariates could be kept during the backward elimination process, and then the CRCL could be removed (data not shown). However, to respect the principle of parsimony, and because serum creatinine (that we observed to have an effect (albeit non-significant) on palbociclib CL/F during the covariate selection process) is also used in the CRCL calculation, we preferred to keep CRCL as the covariate. As expected, keeping only the CRCL compensated for the effect of weight and age on the CL/F, as shown in Figure 2.

It could be argued that CRCL is not a relevant covariate for a drug that is mainly eliminated in feces after extensive metabolism and whose renal clearance accounts for 6.6% to 7.7% of its total clearance [2,5]. However, contrary to covariates associated with the hepatic function (such as AST, ALT or GGT), we observed that only covariates potentially linked with renal clearance were significantly associated with palbociclib CL/F. As we previously shown, if we assume that age and body weight are associated to renal clearance (because they are used to calculate CRCL), such an association with CRCL and palbocilcib CL/F was also highlighted the previous mentioned POP PK study [7]. Similar to our results, these authors also did not observe any link between liver parameters and palbociclib CL/F. A correlation between the CRCL and palbociclib CL/F was also recently described [5]. In this study, the authors observed that mild (60 < CRCL < 89 mL/min), moderate (30 < CRCL < 59 mL/min) or severe (CRCL < 30 mL/min) renal impairment led to an increase of the palbociclib Area Under the Curve (AUC) of 31%, 45% and 57%, respectively, that was relative the geometric mean AUC obtained with subjects with normal renal function. A significantly statistical regression was also described between the palbociclib CL/F and CRCL [5].

Unfortunately, our model was associated with important shrinkage, especially with absorption and error models. However, this is not totally surprising as only one sample was taken per patient. In such a case, shrinkage may happen [8]. In such a situation, individual graphs such as individual predicted versus observed concentrations (IPRED vs. DV) are uninformative. However, the shrinkage associated with the IIV of CL/F was quite low (14.9%). This gives the opportunity to access individual CL/F, and potentially individual exposures, with a certain confidence. Interestingly, such data are obtained with only one sample, without constraints regarding the sampling times and without the need to reach a steady state, as described by the VPC graph. This is all the more important since there is a correlation between palbociclib exposure and the neutrophil count [9]. Our model may therefore be useful to potentially manage neutropenia. However, the data for therapeutic drug monitoring are lacking in such situations. Our model could thus help to collect data with the aim to potentially define the conditions to perform therapeutic drug monitoring of palbociclib.

## 4. Method

### 4.1. Patients and Sampling

We retrospectively reviewed the patient database of our therapeutic drug monitoring platform. All the patients treated with palbociclib from 28 October 2018 to 3 August 2020 were included. All the patients routinely underwent a blood analysis in order to evaluate their palbociclib. All the patients were women receiving palbociclib for breast cancer as part of its indication. The samples were taken during one of the usual follow-up visits. There was no special attention given about either the delay between the administering and the sampling time or about reaching a steady state. However, for each patient, the date and time of the last drug intake and sampling were precisely collected.

No specific informed consent was required as the samples were regular. However, a general consent was signed by each patient stipulating that their data may anonymously be used for research purposes. Thus, the data used in this manuscript were recorded in such a manner that confidentiality was ensured following these guidelines. This protocol was approved by our Institutional Review Board in accordance with the Declaration of Helsinki.

### 4.2. Analytical Methods

The samples were rapidly centrifuged and frozen. They were assayed using a Liquid chromatography-tandem mass spectrometry (LC/MS-MS) validated method, as previously described [10]. Briefly, after alkalinization with NaOH, an extraction was performed with ethyl acetate. After drying, the samples were reconstituted with a mixture of 55% methanol to 45% of the solvent (ammonium acetate 1 M in water, pH adjusted to 3.2 with formic acid). The samples were then filtrated using Captiva ND Lipids plates and 8 µL of the filtrates were injected into the system. The LLOQ for this method was 6 µg/L. The within-run precision (n = 10) and the between-run precision (n = 22) were 8.8% and 9.5%, 9.4% and 0.5% and 4.6% and 3.1%, respectively, at the level of 20 µg/L, 80 µg/L and 480 µg/L, respectively.

### 4.3. Population Pharmacokinetic Analysis

Model development: The model was developed using the non-linear mixed-effects approach. A NONMEM^®^ version 7.4 (ICON Development Solutions, Elliott City, MD, USA) [11] with Pirana interface [12] was used, with the first-order conditional estimation with interaction algorithm. Several absorption processes were tested: first-order absorption with or without lag time or transit compartments. One- and two-compartment models were also assessed. Interindividual variability (IIV) was exponentially modeled.

Covariate selection: Covariates were introduced using a power model and were normalized using the mean value of the parameter as follows:

$$Cl_{i}=Cl_{pop}\times {\left(\frac{COV_{i}}{COV_{mean}}\right)}^{\theta_{COV}}$$

where *Cl_i_* is the individual model-predicted clearance for an individual with a covariate value of *COV_i_*, *COV_mean_* is the mean value of the population for the covariate and *θ_COV_* is the covariate effect. 

For categorical covariates, the parameter value was defined as:

$$Cl_{i}=Cl_{pop}\times \beta^{\theta_{COV}}$$

where *Cl_i_* is the individual model-predicted clearance for an individual, *θ_COV_* is the individual covariate value coded as 0 or 1 and *β* the estimated influential factor of the categorical covariate. The covariate selection was performed by an addition and backward deletion as described in Mould et al. [8], with significant levels for OFV changes of 0.05 (Wald test) for the forward inclusion, and of 0.001 for the backward elimination deletion, in order to account for multiple tests. As the study was conducted in a routine setting, the only easily available covariates were collected. The continuous covariates were age, total body weight, serum creatinine, creatinine clearance (CRCL) calculated following the Cockroft and Gault formula, aspartate (AST) and alanine aminotransferase (ALT), gamma-glutamyl transferase (GGT), alkaline phosphatase, lactate dehydrogenase, total bilirubinemia, serum albumin and total protein concentrations. The categorical covariates were the associated fulvestrant or aromatase inhibitors (letrozole or anastrozole). Only significant covariates were implemented in the final model.

Residual variability: several models of residual error were tested including additional, proportional or combined models.

Model selection: the identification of the best model was based on the objective function value (OFV) for the nested models, or the Bayesian information criterion (BIC) for the non-nested models, and by the inspection of the following diagnostic plots: predicted versus observed concentrations (PRED vs. DV), conditional weighted residuals versus time and predicted concentrations (CWRES vs. TIME and CWRES vs. PRED).

Model assessment: The robustness of the estimates of the final population model was assessed by a bootstrap method [13]. The bootstrap resampling was repeated 500 times, and the 95% confidence intervals (CI95) of the parameters were compared with those obtained from the original dataset. A visual predictive check (VPC-1000 simulations) [14] and normalized prediction distribution errors (NPDE-1000 simulations) [15] were also used for the internal evaluation of the final population model. The bootstrap and VPC were performed with the Perl-speaks-NONMEM (PsN) toolkit [16].

## 5. Conclusions

To our knowledge, we present here the first full-length article describing the POP PK of palbociclib. This model was built with data obtained in a routine setting. This implies that the data were not rich enough to satisfactorily estimate some parameters, especially those of absorption, and that our model was impeded by shrinkage. However, we observed a good estimation of the clearance. Interestingly, this estimation was obtained with only one sample per patient and without care about reaching the steady state. Thus, with a good estimation of the clearance with only one sample, we think that our model may be useful for further studies associated with the PK of palbociclib.

## Figures and Tables

**Table 1:** Characteristics of the patients included in the population pharmacokinetics (POP PK) study of palbociclib in routine settings.

Patient Characteristics	Mean	Median	Min-Max
Age (years)	67.4	70.4	40.7–92.2
Total Body Weight (WT—kg)	69.7	98.0	37.0–140.0
Serum Creatinine (µmol/L)	74.6	68.0	31.0–301.8
Creatinine Clearance—Cockroft & Gault (CRCL—ml/min)	78.9	72.1	23.4–282.3
Alanine aminotransferase (UI/L)	30.7	21.5	13.0–205.0
Aspartate aminotransferase (UI/L)	26.5	16.0	16.0–270.0
γ-glutamyl transferase (UI/L)	74.2	28.5	10.0–1113.0
Alkaline phosphatase (UI/L)	110.9	78.0	11.0–644.0
Lactate dehydrogenase (UI/L)	239.6	223.5	85.0–675.0
Total bilirubinemia (µmol/L)	8.5	7.9	2.7–27.2
Serum albumin (g/L)	39.9	40.0	27.0–47.7
Total protein (g/L)	70.0	70.0	53.0–98.0

**Table 2:** Population-based pharmacokinetic estimates of the parameters of the final model. The estimates are expressed with their relative standard error (RSE) obtained from NONMEM and expressed in percentages and shrinkage for the interindividual variability (IIV) and the errors. The estimates are presented with the median and 2.5th–97.5th percentile values of the bootstrap (500 simulations) for each parameter.

Model Parameters				Bootstrap (n = 500)
	Estimates	RSE	Shrinkage (%)	2.5th–97.5th Percentiles
CL/F (L/h)	58.3	3.3%		54.2–62.8
CRCL on CL/F	0.419	13.9%		0.287–0.560
V/F (L)	1580	16.2%		930–2568
Ka (h^−1^)	0.187	19.3%		0.107–0.370
Tlag (L)	0.658	-		
IIV CL/F	31.3%	36.2%	14.9%	23.5%–36.7%
IIV K_a_	126.1%	36.2%	63.5%	21.2%–259.4%
Correlation between CL/F and K_a_	−34.2%	50.5%		−56.6%–13.8%
Additional Error (µG/L)	8.14	17.6%	58.9%	4.33–14.80
Proportional Error	0.0689	31.3%	58.9%	0.0136–0.135

## Data Availability

Authors can confirm that all relevant data are included in the article.
